# Letts's Medical Diary for 1892

**Published:** 1891-12

**Authors:** 


*Letts's Medical Diary for* 1892. London: CASSELL AND COMPANY, LIMITED.

This *Diary* is convenient in size, is well arranged, and contains much useful information. The list of therapeutic agents, however, is not up to date.

